# Sacituzumab govitecan in metastatic triple-negative breast cancer patients treated at Institut Curie Hospitals: efficacy, safety, and impact of brain metastases

**DOI:** 10.1007/s12282-024-01565-7

**Published:** 2024-04-10

**Authors:** Alexandre De Moura, Delphine Loirat, Sarah Vaillant, Sinen Korbi, Nicolas Kiavue, Diana Bello Roufai, Laurence Escalup, Romain Desmaris, Pauline Vaflard, Paul Cottu, Jean-Yves Pierga, François-Clément Bidard, Luc Cabel, Alexandre Acramel

**Affiliations:** 1 Department of Medical Oncology, Institut Curie, Paris & Saint-Cloud, France; 2 Department of Pharmacy, Institut Curie, Paris & Saint-Cloud, France; 3 UVSQ, Université Paris-Saclay, Saint-Cloud, France; 4 Université Paris Cité, Paris, France; 5 Université Paris Cité, CiTCoM, CNRS UMR 8038, Inserm U1268, Paris, France

**Keywords:** Sacituzumab govitecan, Triple-negative breast cancer, Metastatic breast cancer, Brain metastases

## Abstract

**Background:**

Sacituzumab govitecan (SG) has been approved by FDA in April 2021 for pre-treated metastatic triple-negative breast cancer (mTNBC), following the ASCENT trial results.

**Methods:**

We set up an ambispective bicentric cohort study to assess the real-world effectiveness and safety of SG in patients with mTNBC treated at Institut Curie Hospitals, with a focus on patients with brain metastases.

**Results:**

This study included 99 patients treated through the French Early Access Program to SG from May 2021 to January 2023. Median age was 55 years [26–89], *N* = 8 patients (8%) had *BRCA1/2* mutation, *N* = 12 (12%) de novo stage IV disease and *N* = 31 (31%) brain metastases. Patients had previously received a median of two [1–10] lines of treatment in advanced setting. After a median follow-up of 9.7 months, the median progression-free survival (PFS) and overall survival (OS) were 3.9 months (95%CI[3.4–5.0]) and 8.6 months (95%CI[7.1–11.9]), respectively, while objective response rate was 29% (95%CI[21–39]). Among patients with brain metastases, median PFS and OS were 3.7 months (95%CI[2.6–6.2]) and 6.7 months (95%CI[6.3–NR]), respectively, with intracranial tumor responses. Dose reductions were required in *N* = 17 patients (17%) within a median of three [2–11] cycles, due to gastrointestinal toxicity (*N* = 6; 6%), hematological toxicity (*N* = 9; 9%) including febrile neutropenia (*N* = 2; 2%), liver enzyme elevation (*N* = 1; 1%), and physical deterioration (*N* = 1; 1%). There was no related death to SG.

**Conclusions:**

The observed response rate and safety of SG are consistent with the results of the ASCENT trial, with efficacy observed in patients with brain metastases, but observed PFS and OS are numerically shorter.

## Introduction

Triple-negative breast cancer (TNBC) accounts for 15% of metastatic breast cancers, has a poorer prognosis, and represents a high unmet medical need [1]. Therapeutic progress has been made in recent years, with the established benefit of immunotherapy depending on programmed death-ligand 1 (PD-L1) expression in association with chemotherapy in first-line treatment for advanced disease, but median overall survival remains less than two years [2, 3]. Another avenue of interest is the development of antibody–drug conjugate (ADC).

Sacituzumab govitecan (SG) is an antineoplastic agent, which combines sacituzumab, a humanized monoclonal antibody binding to trophoblast cell-surface antigen-2 (Trop-2)-expressing cancer cells, linked with govitecan (SN38), a topoisomerase I inhibitor. In the phase III ASCENT trial, SG showed a statistically significant progression-free survival (PFS) and overall survival (OS) benefit over chemotherapy (eribulin, vinorelbine, capecitabine, or gemcitabine) for metastatic triple-negative breast cancer (mTNBC) after two or more systemic therapies, with at least one of them in the advanced setting, leading to the Food and Drug Administration (FDA) approval on April 2021 and European Medicines Agency (EMA) approval in November 2021 [4]. The primary end point was PFS among patients without brain metastases, and few patients with brain metastases were treated with SG (*N* = 32). In France, first patients were treated in non-trial clinical practice in May 2021, and access to SG has been expanded in November 2021 through a national early access program (EAP).

In this ambispective analysis of prospectively enrolled patients, we report the outcome and safety of patients treated with SG with and without brain metastases at the Institut Curie Hospitals from May 2021 to January 2023.

## Patients and methods

### Patients and treatment

Eligibility criteria to the French EAP were: age ≥ 18; TNBC (estrogen and progesterone receptors < 10%, HER2-negative, by local assessment); two prior systemic treatments for TNBC with at least one of them in the advanced setting.

All patients treated were prospectively registered. SG was initially started at 10 mg/kg, and dose reductions were possible at the following cycles per SG summary of product characteristics. SG was continued until tumor progression, death, limiting toxicity, or medical or patient decision.

### Data collection

This ambispective study included all consecutive patients and was approved by the Institut Curie review board (DATA230222) in accordance with relevant guidelines and regulations. All patients provided written consent for their clinical data to be reported.

Data were collected regarding patients characteristics (birth date, sex, performance status), tumor characteristics (date of diagnosis, de novo or recurrent metastatic disease, tumor characteristics at the localized stage and at the advanced stage, number and sites of metastases including central nervous system metastases, germline and/or somatic *BRCA* mutation status), medical history (previous systemic lines, previous exposition to anti-PD-1/PD-L1 and PARP inhibitors), information on SG prescription (start and end of treatment date, dosing adjustment, reason for dosing adjustment or discontinuation of treatment), safety (toxicities leading to dosing adjustment or discontinuation of treatment or death), treatment efficacy (with tumor assessment according to RECIST v1.1), and survival data. Data cut-off was January 20th, 2023.

### Endpoints and statistics

First endpoints were progression-free survival (PFS) and overall survival (OS) in the overall population. PFS was the timing from initiation of treatment to the occurrence of disease progression or death, assessed by the physician. Because of its design, this study had no pre-specified power. Secondary endpoints were PFS and OS in patients with brain metastases, objective response rate (ORR, by RECIST criteria v1.1), exploration of prognostic and predictive factors, and safety. The following prognostic factors for PFS were explored: age at baseline, performance status at baseline, previous systemic therapies, triple-negative disease at diagnosis, de novo or recurrent metastatic disease, number and type of metastases (including liver and brain involvement), *BRCA* mutation, prior PARP inhibitor therapy, and anti-PD-1/PD-L1 therapy.

Descriptive statistics were used to summarize the patient characteristics. Survival curves for PFS, median PFS and its 95% confidence interval (95%CI) were generated using the Kaplan–Meier method. Multivariate Cox proportional hazards models were constructed to identify independent prognostic factors. All factors significant at a conservative 5% level in univariate analysis were included in multivariate analysis. All analyses were performed using R version 4.2.2. Statistical significance was defined by a two-tailed *p* < 0.05.

## Results

### Patient characteristics

Individual data of 99 patients treated with SG in the Institut Curie Hospitals between May 2021 and January 2023 are reported. Data were collected until January 20th, 2023. Baseline demographic and clinicopathological characteristics of patients are shown in Table 1. All patients were women, with a median age of 55 years, 12% had de novo metastatic disease, and 31% had brain metastases. On genetic data, 8% had known *BRCA1/BRCA2* mutation: 7 patients had germline mutation and only one had somatic mutation with no associated germline mutation. Patients had previously received a median of two lines [1-10] of treatment in advanced setting, 28% and 6% had previously received anti-PD-1/PD-L1 and PARP inhibitors, respectively. All patients had mTNBC, of whom 26% had non-triple-negative primary breast cancer (estrogen receptor and/or progesterone receptor ≥ 10%) with proven triple-negative metastatic relapse. In this cohort, 48% of the patients met the inclusion criteria for the main published analysis of the ASCENT study.

**Table 1:** Patient characteristics

Characteristic	
Female, *N* (%)	99 (100%)
Age, median (range)	55 (26–89)
*Stage at initial diagnosis, N (%)*	
Stage I–II–III	87 (88%)
Stage IV (de novo metastatic disease)	12 (12%)
*Initial tumor phenotype, N (%)*	
HR-Positive (ER and/or PR ≥ 10%)	26 (26%)
HR-Negative (ER and PR ≤ 10%)	73 (74%)
HER2-Positive	0 (0%)
HER2-Negative	99 (100%)
*Tumor phenotype at SG initiation, N (%)*	
HR-Negative (ER and PR ≤ 10%)	99 (100%)
HR 0 (ER and PR < 1%)	89 (90%)
HR 1–10 (ER or PR > 1% and ≤ 10%)	10 (10%)
*No. of metastatic sites, N (%)*	
1–2	34 (34%)
≥ 3	65 (66%)
Visceral metastases, *N* (%)	80 (81%)
Liver metastases, *N* (%)	44 (44%)
Brain metastases, *N* (%)	31 (31%)
*Performance status, N (%)*^a^	
0–1	79 (80%)
2	17 (17%)
No. of previous lines for metastatic disease, median (range)	2 (1–10)
Prior anti-PD-1/PD-L1 therapy, *N* (%)	28 (28%)
Prior PARP inhibitor therapy, *N* (%)	6 (6%)
*BRCA mutation status, N (%)*	
*BRCA* mutation not tested	7 (7%)
*BRCA* mutation in tumor and/or germline^b^	8 (8%)
No *BRCA* mutation found	84 (85%)
All ASCENT study inclusion criteria met, *N* (%)	48 (48%)

Abbreviations: *HR* hormone receptor, *ER* estrogen receptor, *PR* progesterone receptor, *SG* sacituzumab govitecan

^a^Data on Performance Status was missing for *N* = 3 patients

^b^*N* = 7 patients had *BRCA* germline mutation, *N* = 1 patient had BRCA somatic mutation with no associated germline mutation

### Treatment efficacy and prognostic factors

**Fig. 1:**
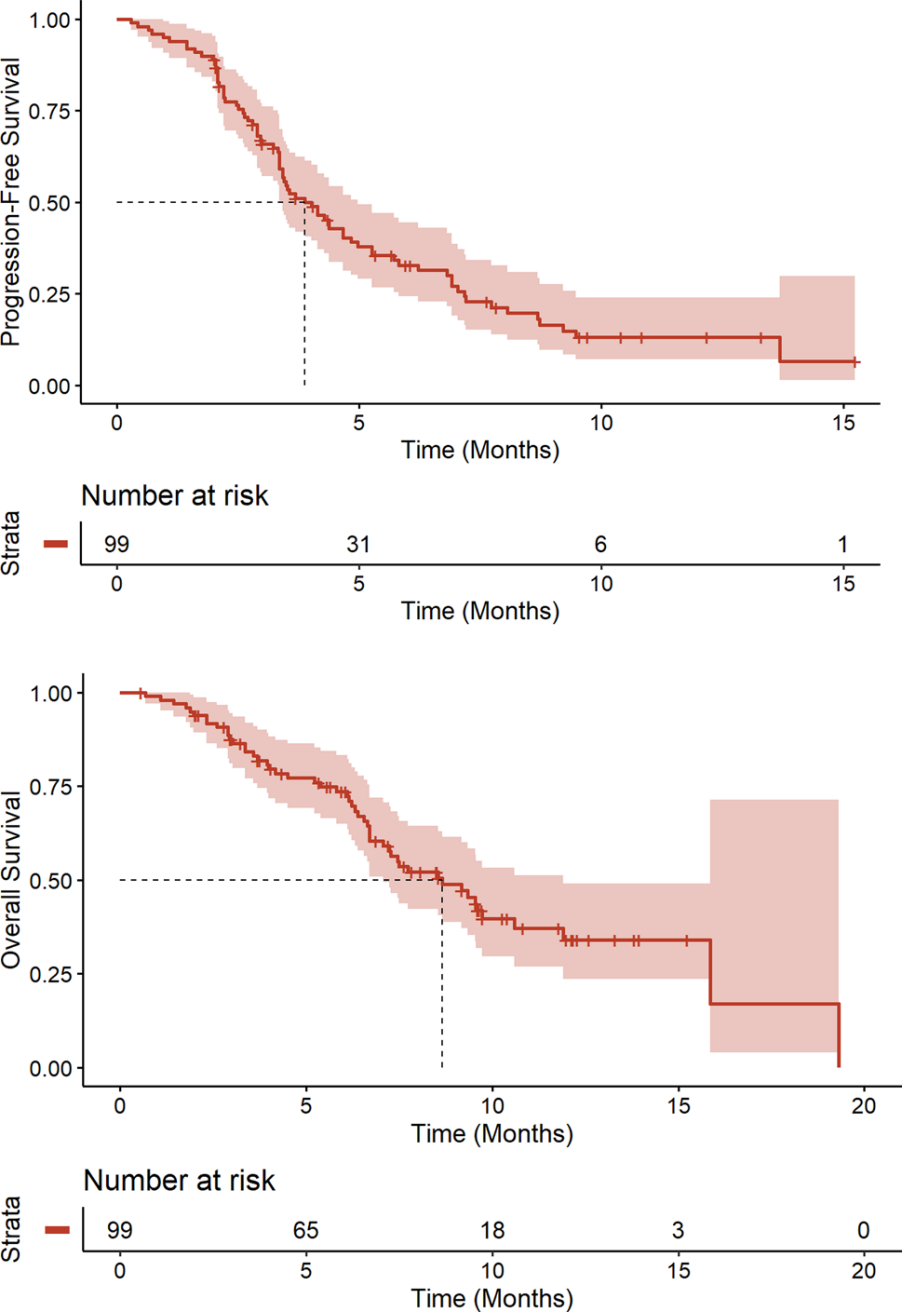
Progression-free survival and overall survival

After a median follow-up of 9.7 months, median PFS was 3.9 months (95%CI[3.4–5.0]) and median OS was 8.6 months (95%CI[7.1–11.9]) (Fig. 1). The ORR was 29% (95%CI[21%–39%]): *N* = 2/99 patients (2%) had a complete response (CR) as best response, *N* = 27/99 (27%) had a partial response (PR), *N* = 23/99 (23%) had a stable disease (SD), *N* = 44/99 (44%) had a progressive disease (PD), and *N* = 3/99 (3%) had a non-evaluable disease. Among patients who met the eligibility criteria for ASCENT study publication (*N* = 48), median PFS was 4.4 months (95%CI[3.5–7.0]) and median OS was 10.6 months (95%CI[9.3–NR]) (Appendix 1).

**Fig. 2:**
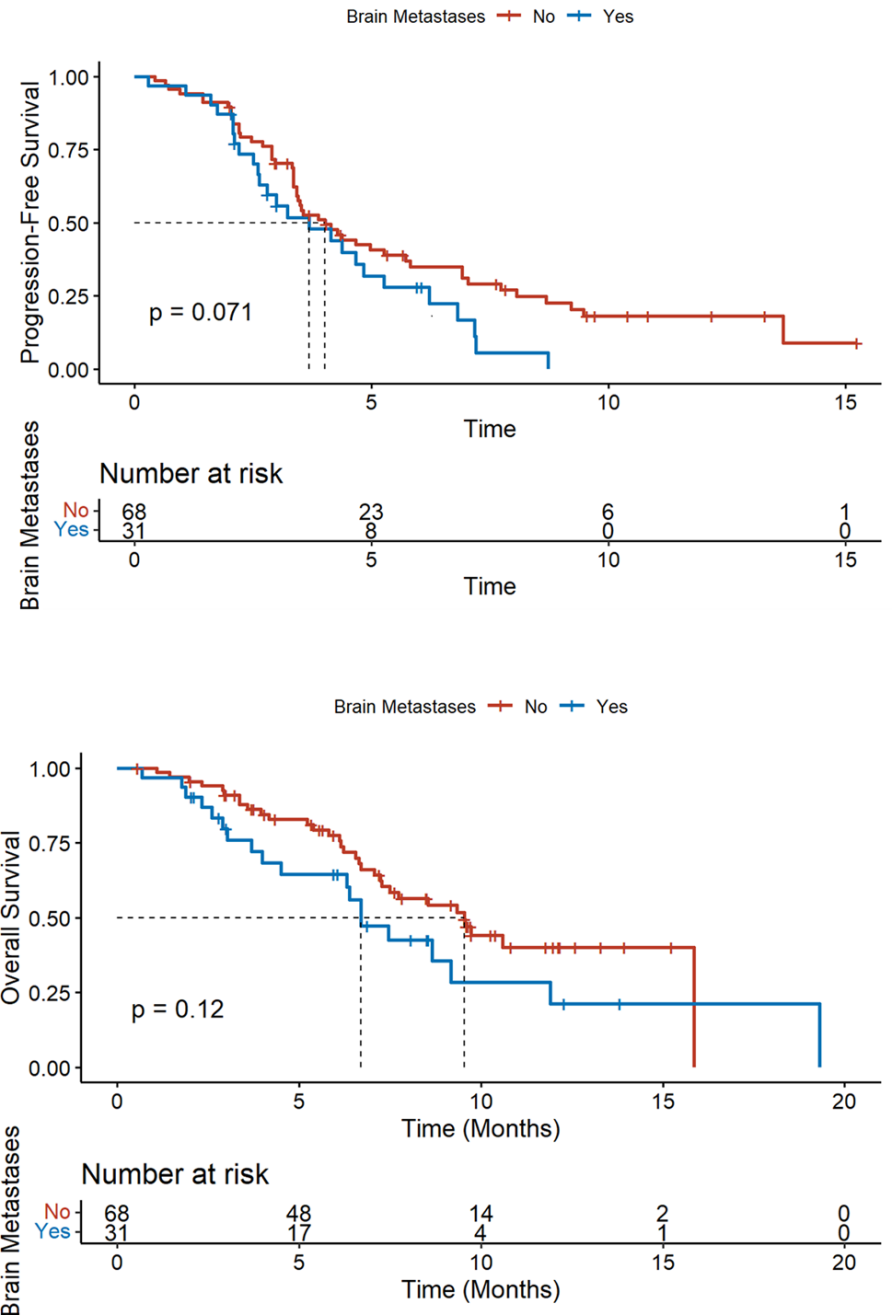
Progression-free survival and overall survival according to brain metastases

**Fig. 3:**
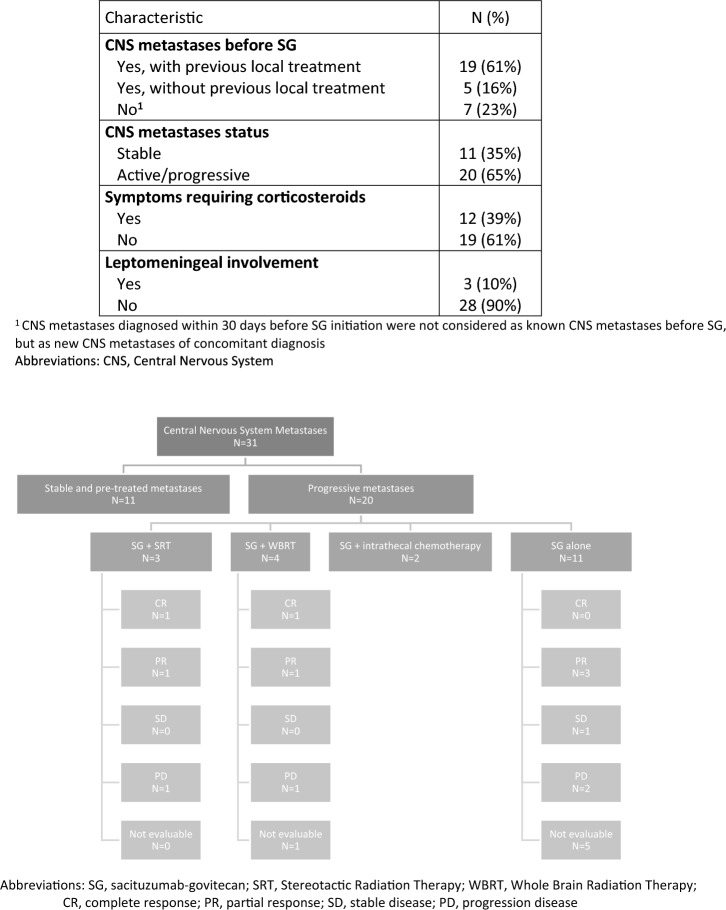
Patients with central nervous system metastases (*N* = 31): description and pattern of response to SG

**Fig. 4:**
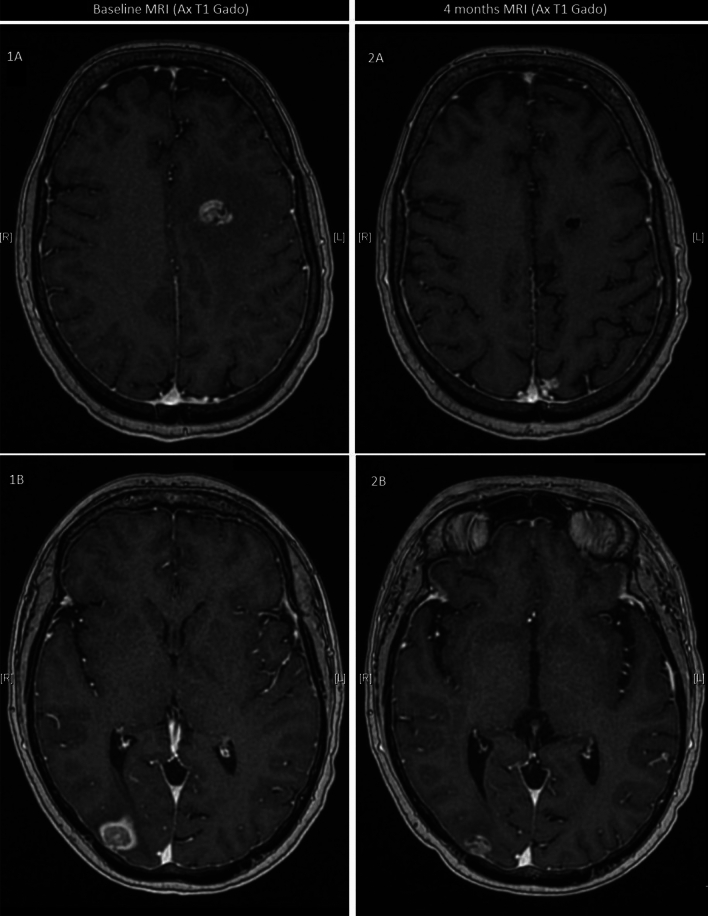
Best intracranial response with sacituzumab govitecan alone (without radiotherapy). Magnetic resonance imaging (MRI), T1 sequence with gadolinium injection. 1A/1B: target brain metastases before SG; 2A/2B: target brain metastases after 4 months with SG alone, without any radiation therapy

Among patients with brain metastases, median PFS was 3.7 months (95%CI[2.6–6.2]) and median OS was 6.7 months (95%CI[6.3–NR]) (Fig. 2). While 11 patients had stable and previously treated brain metastases, 20 had progressive central nervous system metastatic disease before SG administration, of which 11 (55%) were treated with SG alone, 4 (20%) with SG and whole brain radiation therapy (WBRT), 3 (15%) with SG and stereotactic radiation therapy (SRT) and 2 (10%) with SG and intrathecal chemotherapy for leptomeningeal metastases (methotrexate) (Fig. 3). Six patients treated with SG alone had radiologically evaluable cerebral disease according to RECIST 1.1, 50% (*N* = 3/6) had a partial intracranial response. The best intracranial response is shown in Fig. 4. We could not report intracranial PFS due to an inconsistent and heterogeneous monitoring. Among the 6 patients treated with SG and local treatment with evaluable cerebral disease, *N* = 2/3 patients had intracranial partial response with SG + WBRT (one could not be evaluated) and *N* = 2/3 patients had intracranial partial response with SG + SRT. We cannot report an ORR for the two patients treated with concomitant SG and intrathecal methotrexate for leptomeningeal disease, but the first patient had clinical progression leading to death after one cycle, and the second achieved a complete cytological and biochemical response assessed on cerebrospinal fluid, resulting in the control of the leptomeningeal disease for 4 months.

Of the 99 treated patients, 75 (76%) discontinued SG due to progressive disease (*N* = 70; 93%), toxicity (*N* = 1; 1%), physical deterioration (*N* = 3; 4%), or the patient’s request (*N* = 1; 1%). Twenty-four patients were on treatment at data cut-off. The median duration of treatment was 3.4 months, corresponding to 5 cycles of SG. Of the 75 patients who discontinued SG, 25% (*N* = 19) received exclusive palliative care and 75% (*N* = 56) received further anti-tumor treatment: the next line was mainly standard chemotherapy (*N* = 48), some patients were included in a clinical trial (*N* = 6) or received PARP inhibitor (*N* = 1) or another ADC (*N* = 1) (Appendix 2).

**Fig. 5:**
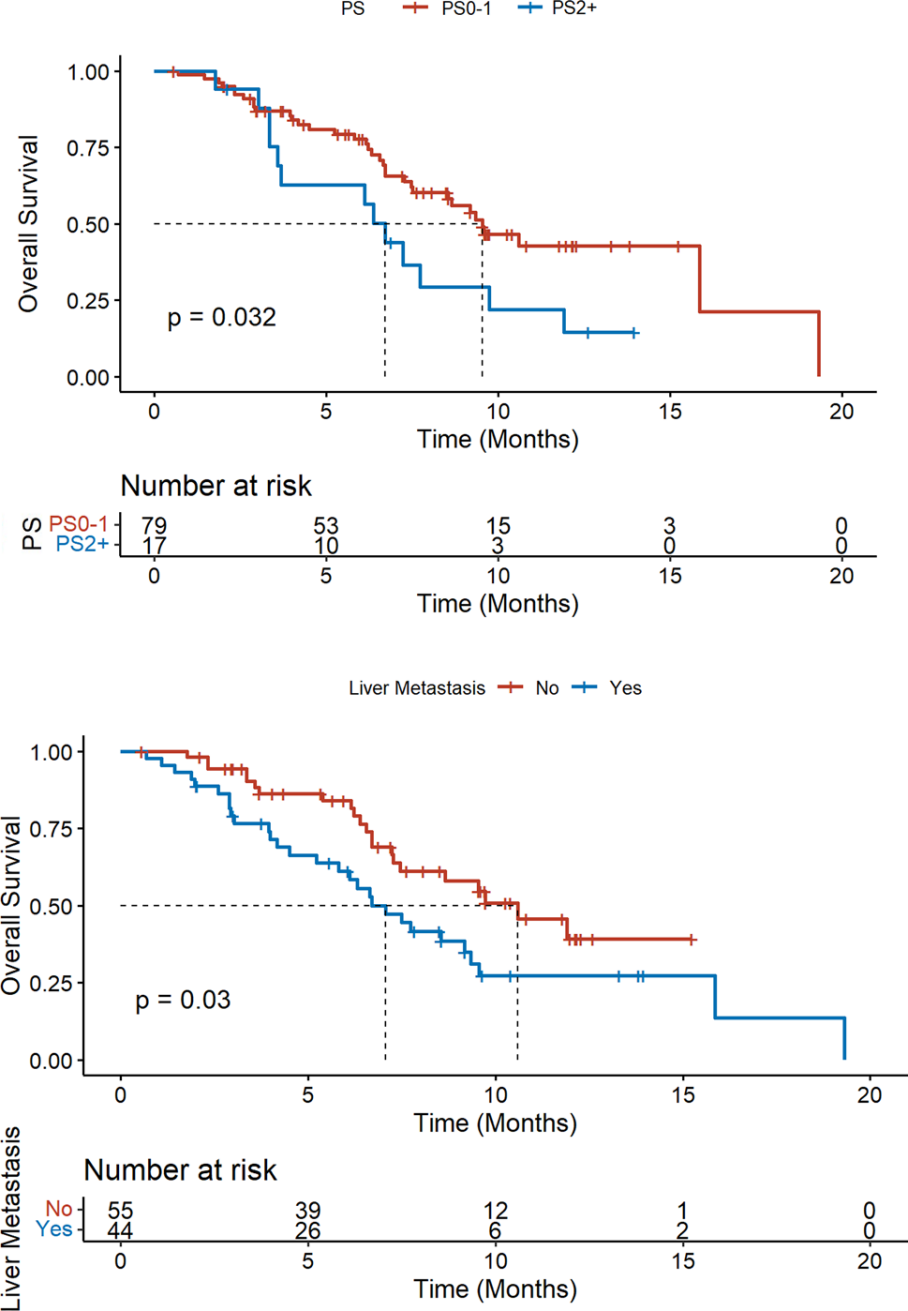
Overall survival according to performance status and liver metastases

Univariate and multivariate analyses were undertaken to explore the factors associated with PFS and OS (Table 2). We reported no subgroup with significant better PFS. An altered PS (≥ 2) and liver metastases were associated with lower OS in univariate analysis (HR 2.0, 95%CI[1.0–3.8] and HR 1.8, 95%CI[1.1–3.3], respectively), confirmed in multivariate analysis (HR 2.2, 95%CI[1.2–4.3] and HR 2.0, 95%CI[1.1–3.6], respectively) (Fig. 5).

**Table 2:** Exploration of prognostic factors associated with progression-free survival and overall survival

Characteristic	HR PFS (95%CI) univariate	HR OS (95%CI) univariate	HR OS (95%CI) multivariate
Age	1.0 (0.99–1.0)	1.0 (0.98–1.0)	
Stage at initial diagnosis: IV (de novo mTNBC)	1.5 (0.75–2.9)	1.5 (0.63–3.6)	
Initial tumor phenotype: TNBC	0.79 (0.47–1.3)	0.77 (0.41–1.4)	
Tumor phenotype at SG initiation: HR 1–10	0.72 (0.29–1.8)	1.5 (0.52–4.1)	
No. of metastatic sites: ≥ 3	1.6 (0.96–2.6)	1.4 (0.76–2.6)	
Visceral metastases	1.7 (0.86–3.3)	2.3 (0.92–5.9)	
Liver metastases	1.3 (0.85–2.1)	**1.8 (1.1–3.3)**	**2.0 (1.1–3.6)**
Brain metastases	1.6 (0.95–2.6)	1.6 (0.89–2.8)	
Performance Status: ≥ 2	1.7 (0.95–2.9)	**2.0 (1.0–3.8)**	**2.2 (1.2–4.3)**
No. of previous lines for metastatic disease: ≥ 3	1.6 (0.99–2.5)	1.7 (0.98–3.0)	
Prior anti-PD-1/PD-L1 therapy	1.2 (0.72–1.9)	0.9 (0.49–1.7)	
Prior PARP inhibitor therapy	1.3 (0.54–3.3)	1.0 (0.30–3.4)	
*BRCA* mutation	0.86 (0.35–2.2)	0.97 (0.30–3.1)

### Safety

Out of 99 treated patients, one experienced toxicity leading to SG discontinuation (grade 4 neutropenia). Dose reductions were required in 17 patients (17%) within a median of 3 cycles [2-11]. The main limiting toxicity was hematological (*N* = 9) including neutropenia (*N* = 6), febrile neutropenia (*N* = 2), and anemia (*N* = 1). Limiting gastrointestinal toxicity was also common (*N* = 6) with diarrhea grade 2 (*N* = 1) or 3 (*N* = 5) which could be associated with radiological colitis (*N* = 2). Other toxicities were liver enzyme elevation (*N* = 1) and physical deterioration (*N* = 1). There was no related death to SG.

## Discussion

Our real-world data in mTNBC patients are consistent with results of the ASCENT trial in terms of ORR, which was 29% in our cohort and 35% in the ASCENT publication, but observed PFS and OS were numerically shorter.

In the ASCENT trial, median PFS was 5.6 months versus 3.9 months in our cohort whereas median OS was 12.1 months versus 8.6 months in our cohort. Results in this real-life cohort are numerically shorter but remain superior to the standard chemotherapy arm in the ASCENT trial. Those shorter PFS and OS in real-life data are common with a less selected population: the main differences in our cohort were the 17% of patients with altered PS which were not eligible in the ASCENT trial and the 31% of patients with brain metastases which were excluded from the main analysis of the ASCENT trial, these two factors being known detrimental prognostic factors [5–7]. The negative prognostic impact of these factors was also suggested by the additional analysis we carried out by excluding patients who did not meet the eligibility criteria for the ASCENT study, as well as patients with controlled brain metastases who were eligible for the ASCENT study but whose data were not published in the main analysis. In this subgroup of 48 patients, survival data were numerically better and were close to those of the ASCENT study with a median PFS of 4.4 months and a median OS of 10.6 months. The exclusion of 52% of our cohort for this comparative analysis with ASCENT study clearly illustrates the difference between real-life populations and clinical trial populations and the relevance of publishing real-life data.

We note that the proportion of patients with initial triple-negative disease in our cohort is in line with the ASCENT population, and that published data supports SG efficacy for mTNBC regardless of subtype at initial diagnosis [8]. However, we must point out differences in the definition of triple-negative disease in our cohort compared to ASCENT study. The threshold for ER expression and PR expression in our cohort was < 10%, based on European and French guidelines, and as allowed in the French early access program. This threshold differs from the inclusion criteria of the ASCENT study (ER and PR < 1% based on ASCO guidelines), with *N* = 10 (10%) additional patients treated and no evidence of reduced efficacy in these patients, although the statistical power is insufficient to conclude in this small subgroup.

Although patients with controlled brain metastases were eligible for the ASCENT study, they only represented 12% of the enrollment, with 32 patients in the SG arm. In those patients, the median PFS was 2.8 months (95%CI[1.5–3.9]) and median OS was 6.8 months (95%CI[4.7–14.1]) [9]. SG was numerically better than standard chemotherapy for tumor response and PFS without OS benefit, but data interpretation is limited by the small sample size of patients with brain metastases. In our cohort of 31 patients with active (*N* = 20) or stable (*N* = 11) brain metastases, median PFS and OS were 3.7 months and 6.7 months, respectively. We report the first data on patients with progressive brain metastases treated with SG. Interestingly, we observed 3 cases of partial objective response with SG alone out of 6 radiologically evaluable patients, confirming that SG has its own intracranial anti-tumor activity. Regrettably, the intracranial effect of SG was not evaluable in most of the population because of confounding concomitant treatments (radiotherapy, intrathecal chemotherapy) or disease that was not radiologically assessable or that have not been re-evaluated in a retrospective context. ADCs could be a way of improving the control and response of intracranial metastases, as reported with trastuzumab deruxtecan in HER2-positive breast cancer [10], but few data are available with SG and no data prior to our study had reported to our knowledge an objective intracranial response with SG in TNBC [11]. Those findings confirm that SG could be considered for patients with active brain metastases, in absence of available local treatment, but SG efficacy on active brain metastases should be evaluated in dedicated further studies. We also describe the first data from a cohort of 7 patients treated with SG and radiotherapy, with only one case report published to date [12]. Objective responses have been observed, probably mainly related to radiotherapy, without knowing the contribution of SG to these intracranial responses. In our cohort, no cerebral radiotherapy was carried out concomitantly with SG due to lack of data about the safety. A short therapeutic window was made in the context of a mostly rapidly progressive disease: this was reduced to a minimum of 5 days between SG and radiotherapy, corresponding to at least 5 times the half-life of the drug, with no significant toxicity reported. Data suggest an increased incidence of radionecrosis with concomitant use of others ADC such as trastuzumab deruxtecan, calling for vigilance [13]. Concomitant encephalic and extraencephalic progression is frequent in current practice in mTNBC patients: additional data and specific studies on the association between SG and radiotherapy are required.

The toxicity profile of SG in our real-life cohort is consistent with data from ASCENT trial, with adverse events leading to SG dose reduction in 17% of patients and SG discontinuation in only one patient. Main toxicities were gastrointestinal and hematological known toxicities, routinely manageable with prophylactic or curative treatments and dose adaptation. No signal for a new toxicity has appeared in our cohort.

This analysis was performed on consecutive patients receiving SG as part of the French EAP, which allows the early evaluation of new drugs in a ‘real-life’ context [14, 15]. Finally, our report supports the benefit and safety of SG in the treatment of mTNBC patients, including those with brain metastases. Since it is a two-center cohort study in France, it cannot be directly translated to other cohorts with different healthcare systems and patient characteristics, and it would be interesting to have additional real-life data from other countries.

## Data Availability

This manuscript has no associated data or the data will not be deposited (data is available upon request from the Authors).

## Appendix 2. Subsequent therapies in patients who discontinued SG (*N* = 75)

**Table:**

Anticancer therapy	*N* (%)
Exclusive palliative care	19 (25%)
Standard chemotherapy	48 (64%)
Eribulin	20 (27%)
Pegylated liposomal doxorubicin	7 (9%)
Vinorelbine	6 (8%)
Carboplatin	4 (5%)
Capecitabine	3 (4%)
Docetaxel	2 (3%)
Doxorubicin + cyclophosphamide	2 (3%)
Carboplatin + etoposide	1 (1%)
Carboplatin + paclitaxel	1 (1%)
Gemcitabine	1 (1%)
Oral metronomic cyclophosphamide	1 (1%)
Inclusion in a clinical trial	6 (8%)
Antibody drug conjugate	1 (1%)
Trastuzumab deruxtecan	1 (1%)
PARP inhibitor therapy	1 (1%)
Olaparib	1 (1%)

## Code availability

The manuscript has no associated code/software.
